# Cardiovascular magnetic resonance imaging findings in Ebstein anomaly

**DOI:** 10.1186/1532-429X-14-S1-P107

**Published:** 2012-02-01

**Authors:** Nelsy C González, Luis Muñoz, Gabriela Meléndez, Erick Alexánderson, Sergio G Olmos, Juan M Bonelli, Aloha Meave, Luis Marroquin, Fernando Iñarra, José E Telich-Tarriba

**Affiliations:** 1 Cardiovascular Magnetic Resonance Imaging, National Institute of Cardiology "Ignacio Chávez", Mexico City, Mexico; 2 Embryology, National Institute of Cardiology "Ignacio Chávez", Mexico City, Mexico; 3 Nuclear Cardiology, National Institute of Cardiology "Ignacio Chávez", Mexico City, Mexico

## Background

In recent years, cardiac magnetic resonance (CMR) imaging has emerged as the reference standard for cardiac imaging in patients with many forms of congenital heart disease, in particular for lesions that affect the right ventricle. Few studies have been published in assessment of Ebstein anomaly by CMR imaging.

## Methods

CMR studies of patients with EA conducted from June 2007 to June 2011 were analized. Cine spin echo images in multiple views, and angiography were evaluated to assess chambers morphology and diameters, wall thickness and left ventricular function, also were measured the atrialized right ventricular portion and the functional tricuspid valve position. And Inversion Recovery sequence for determination of fibrosis.

## Results

Ninety four patients were studied. Table 1 summarizes the main results. Tricuspid valve regurgitation was found in 96.9%, severe in 62. 5% of the cases. The presence of left ventricle fibrosis was significant associated with right ventricle longest dimension including the atrialized portion (p=0.003), the diastolic and systolic diameters (p=0.011 and p=0.005).

**Table 1:** Results

Variable	Mean	SD
Age (years)	23.0	13.02231
LVdV (mm)	36.5	8.36997
LVsD (mm)	25.7	6.55335
dS (mm)	7.9	10.5891
Lateral wall diastolic (mm)	6.2	1.42729
RVdD (mm)	55.6	20.61004
RVsD (mm)	47.6	21.16745
Right ventricular longitudinal diameter (mm)	96.1	22.96363
RVfw (mm)	4.5	1.40918
RA U/L (mm)	66.0	22.36908
RA M/L (mm)	63.9	20.99671
LA U/L (mm)	44.1	16.28296
LA M/L	36.6	14.03464
LVEF (%)	47.2	10.96851
LV mass (gr)	60.0	28.60894
LVEDV (ml)	78.1	34.64511
LVESV (ml)	40.7	18.73527
Left ventricular stroke volume (ml)	36.6	19.18734
Atrialized portion of right ventricle (mm)	56.1	26.3945
Atrialized portion of right ventricle (%)	59.3	19.84599
Atrial septal defect (mm)	5.7	6.98696
Pulmonary artery (mm)	19.6	6.87248
Right pulmonary branch (mm)	12.8	4.50256
Left pulmonary branch (mm)	13.3	4.49312

LVdD: Left ventricular diastolic diameter; LVsD: Left ventricular systolic diameter; dS: diastolic septum; RVdD: right ventricular diastolic diameter; RVsD: right ventricular systolic diameter; RVfw: right ventricular free Wall; RA U/L: right atrium upper/lower; RA M/L: right atrium mid/lateral; LA U/L: left atrium upper/lower; LA M/L: mid/lateral; LVEF: ejection fraction; LV: left ventricular; LVEDVI: end-diastolic volume; LVESVI: end-systolic volume.

## Conclusions

CMR findings in EA were demonstrated. There was a clear positive relationship between the presence of LV fibrosis and the right ventricle dimensions.

## Funding

National Institute of Cardiology "Ignacio Chávez", Mexico City, Mexico.

